# FRAT-up, a Web-based Fall-Risk Assessment Tool for Elderly People Living in the Community

**DOI:** 10.2196/jmir.4064

**Published:** 2015-02-18

**Authors:** Luca Cattelani, Pierpaolo Palumbo, Luca Palmerini, Stefania Bandinelli, Clemens Becker, Federico Chesani, Lorenzo Chiari

**Affiliations:** ^1^Department of Electrical, Electronic, and Information Engineering - DEIUniversity of BolognaBolognaItaly; ^2^Geriatric UnitAzienda Sanitaria FirenzeFirenzeItaly; ^3^Robert-Bosch-Krankenhaus Geriatric Rehabilitation ClinicStuttgartGermany; ^4^Department of Computer Science and Engineering - DISIUniversity of BolognaBolognaItaly

**Keywords:** accidental falls, odds ratio, risk assessment, risk factors, ROC curve, aged

## Abstract

**Background:**

About 30% of people over 65 are subject to at least one unintentional fall a year. Fall prevention protocols and interventions can decrease the number of falls. To be effective, a prevention strategy requires a prior step to evaluate the fall risk of the subjects. Despite extensive research, existing assessment tools for fall risk have been insufficient for predicting falls.

**Objective:**

The goal of this study is to present a novel web-based fall-risk assessment tool (FRAT-up) and to evaluate its accuracy in predicting falls, within a context of community-dwelling persons aged 65 and up.

**Methods:**

FRAT-up is based on the assumption that a subject’s fall risk is given by the contribution of their exposure to each of the known fall-risk factors. Many scientific studies have investigated the relationship between falls and risk factors. The majority of these studies adopted statistical approaches, usually providing quantitative information such as odds ratios. FRAT-up exploits these numerical results to compute how each single factor contributes to the overall fall risk. FRAT-up is based on a formal ontology that enlists a number of known risk factors, together with quantitative findings in terms of odds ratios. From such information, an automatic algorithm generates a rule-based probabilistic logic program, that is, a set of rules for each risk factor. The rule-based program takes the health profile of the subject (in terms of exposure to the risk factors) and computes the fall risk. A Web-based interface allows users to input health profiles and to visualize the risk assessment for the given subject. FRAT-up has been evaluated on the InCHIANTI Study dataset, a representative population-based study of older persons living in the Chianti area (Tuscany, Italy). We compared reported falls with predicted ones and computed performance indicators.

**Results:**

The obtained area under curve of the receiver operating characteristic was 0.642 (95% CI 0.614-0.669), while the Brier score was 0.174. The Hosmer-Lemeshow test indicated statistical significance of miscalibration.

**Conclusions:**

FRAT-up is a web-based tool for evaluating the fall risk of people aged 65 or up living in the community. Validation results of fall risks computed by FRAT-up show that its performance is comparable to externally validated state-of-the-art tools. A prototype is freely available through a web-based interface.

**Trial Registration:**

ClinicalTrials.gov NCT01331512 (The InChianti Follow-Up Study); http://clinicaltrials.gov/show/NCT01331512 (Archived by WebCite at http://www.webcitation.org/6UDrrRuaR).

## Introduction

### Background

About 30% of community-dwelling people aged 65 or more experience at least one unintentional fall a year [1], and the annual fall rate in this cohort is about 0.65 falls per person [2]. Falls can result in injuries and are a leading cause of activity restriction, hospitalization, and disability [3,4]. Falling is the tenth leading cause of global years lived with disability (YLD). Worldwide, it accounts for about 20 million YLD [5] and a total of 35 million disability-adjusted life years [6]. Its burden is even more pronounced in countries with an older population; in Italy it is estimated to be the third leading cause of YLD [7].

Many preventive strategies have been proposed, and some of them have been shown to be effective [8-10]. Their implementation, however, has been slow and the coverage in Europe is insufficient [11-13]. The individual and societal costs of these interventions are often among the factors that hinder their implementation. In order to make use of available resources and intervene only with subjects at increased risk, medical associations and national health authorities recommend the adoption of fall-risk assessment tools [14-17].

### Existing Tools

Reviews of fall-risk assessment tools and their accuracy are available in the literature [18-23]. Among the most used and validated tools are the Timed Up and Go Test (TUG), the Performance Oriented Mobility Assessment (POMA), and the Physiological Profile Assessment (PPA) [24]. Despite extensive research, existing assessment tools for fall risk have been insufficient for predicting falls [23,25-28].

### Existing Knowledge and Ontologies

An impressive number of scientific publications have identified statistical correlation between the exposure to risk factors and the risk of falling, in terms of odds ratios. Moreover, several reviews and meta-analyses are available, thus providing a solid scientific base about fall-risk factors [29-35].

In our Fall-Risk Assessment Tool (FRAT-up), we faced the issue of representing the information available from scientific literature in a structured manner. In computer science, an ontology is a formal, explicit specification of a shared conceptualization [36]; ontologies are widely used in artificial intelligence, the semantic Web, and biomedical informatics as a form of knowledge representation. Formal approaches, like ontologies and the semantic Web, are important instruments also in epidemiology research [37].

### Aims of the Study

The goal of FRAT-up is to provide a tool for the fall-risk assessment of subjects aged 65 or up and living in a community dwelling. The tool is mainly intended for two different health professional roles: (1) general practitioners (GPs) delivering primary care provisions, with no specific knowledge about falls, who need an assessment tool for evaluating subjects’ fall risk and possible early interventions, and (2) professionals involved in fall prevention and treatment, who need a tool for constantly assessing the fall risk in a reliable and quantitative manner. We identified the following requirements: (1) the assessment tool should identify people at high risk of falling, (2) the tool should exploit existing knowledge about fall-risk factors, (3) the tool should be sufficiently flexible to allow the use of different clinical tests for the estimate of each risk factor, and (4) the assessment tool should be robust with respect to the unavailability of complete information about the subject.

FRAT-up has been developed within the FARSEEING Project [38], and it aims to meet all the requirements listed above.

## Methods

### Overview

The FRAT-up fundamental hypothesis is to consider the fall risk as being directly related to the subject’s exposure to known risk factors. Thus, the starting point is the scientific literature that lists risk factors, together with quantitative information on their association with falls (usually in terms of odds ratios). However, such literature does not provide any structured definition of risk factors and related information. Hence, the first building block of the FRAT-up approach consists of a formal ontology listing risk factors and related data.

Once quantitative information is available through the FRAT-up risk factor ontology, we need to decide how (the exposure to) each risk factor contributes to the overall risk. Our approach is based on probabilities, while epidemiological studies on risk factors usually provide information in terms of odds ratios. Hence, the second building block is a mathematical transformation from odds ratios to probabilities under a few assumptions, as explained further in this section.

The third building block of FRAT-up is a Logic Programming with Annotated Disjunctions (LPAD) program that allows representation of the contribution of each risk factor in terms of probabilistic rules and probabilistic reasoning.

### A Formal Ontology for Fall-Risk Factors

**Figure 1:**
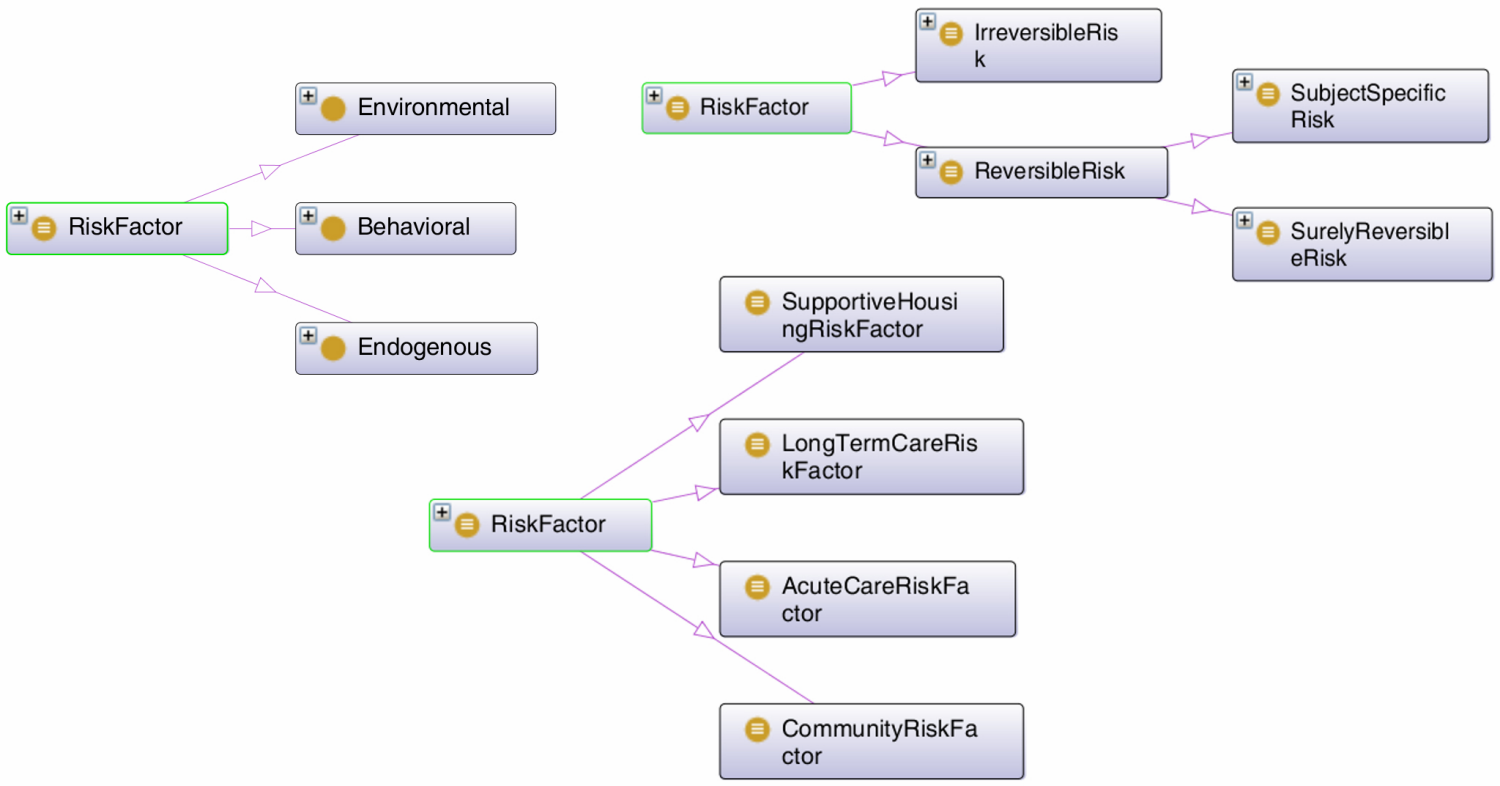
Classification of risk factors by kind, reversibility, and setting. While the InCHIANTI dataset is about community dwellings, the ontology covers other settings, too.

In FRAT-up, a fall-risk factor ontology has been defined, taking into account several domains. For example, the classification of risk factors by reversibility (surely reversible, subject specific reversible, or irreversible) and setting (community dwelling, acute care, etc) is shown in Figure 1.

Within the ontology, risk factors are distinguished as *dichotomous*, *scalar,* and *synergy* factors. Dichotomous risk factors indicate whether a risky condition is present or not, without taking into account its severity. Scalar risk factors also indicate the magnitude of the subject’s exposure to the risky condition. Since synergism between risk factors is well known [39], synergy factors make it explicit if two or more risk factors, due to their simultaneous presence, determine a higher risk than if present alone.

The set of risk factors we include in the ontology comes from a well-established meta-analysis on known risk factors for falls in community-dwelling older people by Deandrea et al [29] (see Table 1).

The ontology also includes the odds ratio for each risk factor, taken from Deandrea et al [29]. Moreover, we introduced in the ontology a clear distinction between a risk factor and the corresponding *estimators*. An estimator is a method to assess the presence and, when necessary, the severity of a risk factor (possibly in combination with other estimators).

Additional data contained in the ontology are the risk factors’ prevalence and procedures to map estimators into factors. Complete information, including sources for quantitative data, is reported in Multimedia Appendix 1.

**Table 1:** Risk factor names and types.

Name	Type
age	scalar
cognition impairment	dichotomous
depression	dichotomous
diabetes	dichotomous
comorbidity	synergy
dizziness and vertigo	dichotomous
fear of falling	dichotomous
female sex	dichotomous
gait problems	dichotomous
hearing impairment	dichotomous
history of falls	dichotomous
history of stroke	dichotomous
instrumental disability	dichotomous
living alone	dichotomous
number of medications	scalar
pain	dichotomous
parkinson	dichotomous
physical activity limitation	dichotomous
physical disability	dichotomous
poor self-perceived health status	dichotomous
rheumatic disease	dichotomous
urinary incontinence	dichotomous
use of antiepileptics	dichotomous
use of antihypertensives	dichotomous
use of sedatives	dichotomous
vision impairment	dichotomous
walking aid use	dichotomous

### From Odds Ratios to Probabilities

#### Overview

The FRAT-up risk-assessment algorithm is based on probability contributions from single risk factors. In the following, we show how we extract probabilities from odds ratios by means of a few mathematical steps.

Initially, we assume that each risk factor is dichotomous; we explain this further in the section on how to generalize to cases with scalar and synergy risk factors. Let *E*_0_, *E*_1_,…, *E*_*n*_ be *n* + 1 dichotomous random variables with values in {0;1}, and *E=*(*E*_0_, *E*_1_,…, *E*_*n*_). We say that the *i* ^*th*^ risk factor is present if *E*_*i*_=1. Let *d*_0_, *d*_1_,…, *d*_*n*_ be *n* + 1 events. We assume the following conditional independence relations:

Equation 1: *d*_*i*_ | *E*_*i*_ ⊥ *d*_*j*_, *E*_*j*_ ∀*j* ≠ *i*

**Figure 2:**
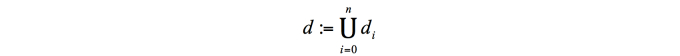
Definition of fall event.

We call *d*_*i*_ a fall event specific to risk factor *E*_*i*_. Assumptions from Equation 1 can be phrased saying that risk factor–specific falls are mutually independent conditional on their associated risk factor. We define the event *d* as the union of the factor-specific events, *d*_*i*_’s (Figure 2). That is, *d* is verified if at least one of the *d*_*i*_’s is verified. This is an assumption of causal independence where the “causes”, *E*_0_, *E*_1_,…, *E*_*n,*_ contribute independently to the probability of the effect *d*; for a complete formal definition see [40]. In our case study, *d* is the presence of at least one fall event during a given time span (if there is no fall, it is not verified), while *E* is an observation of the risk factor exposures of a subject before the time span.

**Figure 3:**
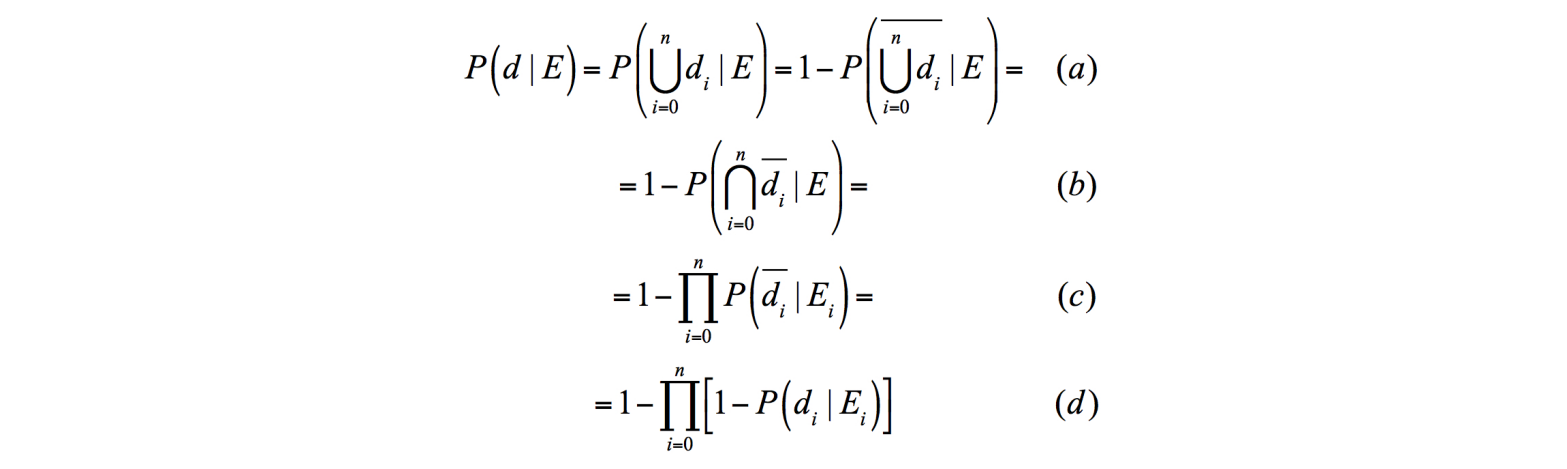
Probability to fall from risk factor specific probabilities.

**Figure 4:**
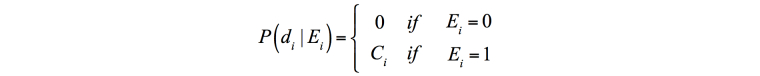
Probability of factor specific fall event given exposure.

**Figure 5:**
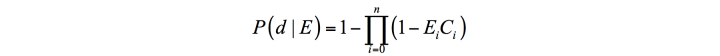
Probability to fall given exposures and contributions.

**Figure 6:**
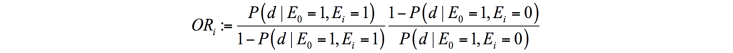
Odds ratio definition.

The conditional probability of *d* given *E* can then be calculated as in Figure 3, by De Morgan laws and assumptions in Equation 1. This function models the probability of an event given a set of possible causes and is known as noisy-OR gate [41] (in this case OR refers to the logical operator). We make the assumption in Figure 4. *C*_*i*_ is a quantity yet to determine. *C*_*i*_ is the contribution to the probability of the effect *d* given by the exposure to the risk factor *E*_*i*_. A method to assign values to the contributions *C*_*i*_ is introduced in the following. Using the equation in Figure 4, the equation in Figure 3 becomes the one depicted in Figure 5. Since we want to model a minimum probability of the adverse event that is applied even in the absence of any observation-specific exposures, we assign *P*(*E*_0_=1)=1. *C*_0_ is the risk that is present in this case. To assign values to the contributions of the exposures, we start from the OR. The OR relative to risk factor *E*_*i*_, with *i*=1,…, *n*, is defined as in Figure 6. Note that the condition *E*_0_=1 is always true and is highlighted above just for convenience.

There is no single way of translating odds ratios to probabilities, since an exact function would require more information than what is conveyed by the odds ratios alone, so some assumptions are needed. We present a possible set of assumptions that leads to a univocal way of computing exposure contributions.

#### Assumption (a)

**Figure 7:**
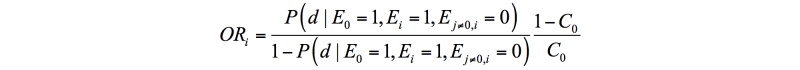
Approximated odds ratio.

We assume that *OR*_*i*_ may be approximated as in Figure 7. Informally, Assumption (a) states that the odds ratio computed on the whole population is similar to the odds ratio computed restricting the population to subjects having at most one exposure. This assumption is obviously true in models where each subject has at most one exposure; otherwise there is a difference in the two values. This has not been quantified yet; the quality of the approximation will be experimentally compared with other methods as a future development.

**Figure 8:**
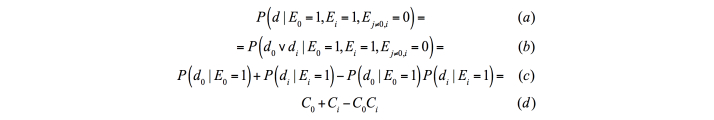
Probability to fall with exposure to exactly 1 risk factor.

**Figure 9:**
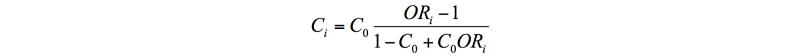
Contribution to fall probability from exposure to a single risk factor given odds ratio.

**Figure 10:**
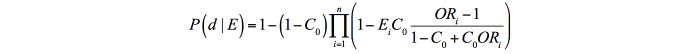
Probability to fall from risk factor odds ratios.

Given the assumptions in Equation 1 and Figure 4, the derivation depicted in Figure 8 follows. Substituting the equation in Figure 8 in the equation in Figure 7 and solving for *C*_*i*_, we finally get the equation depicted in Figure 9. We substitute it in the equation in Figure 5, with a result that is depicted in Figure 10.

#### Assumption (b)

We assume to know *C*_0_, which was calculated by leaving it as a free parameter and then learning it with an equation-solving algorithm. In particular, we used the bisection method, imposing the reported number of total falls from [1].

This model requires that we know for every risk factor if it is present or not. In the following section, we present the way FRAT-up deals with missing values.

For a general reference on how to get relative risk from odds ratio and the incidence of the outcome of interest in the unexposed group, see [42].

#### LPAD Structure and Handling of Unknown Exposures, and Scalar and Synergy Risk Factors

LPADs are logic programs [43] where the head of a clause is a disjunction of annotated atoms. The clauses are of the form:

*h*_1_: *p*_1_v … v *h*_*n*_: *p*_*n*_← *b*_1_∧ … ∧ *b*_*m*_∧ ⌉*c*_1_∧ … ∧ ⌉*c*_*l*_

where *h*_1_,…, * h*_*n*_ are the atoms, and * p*_1_,…, * p*_*n*_ are the probabilities related to each disjunct. Each atom *h*_*i*_ has probability *p*_*i*_ if the body is true, and the atom does not appear in the head of any other clause. When it does, the intended semantics are the distribution semantics as in [44], with the bodies contributing independently to the probability of the atom [40]. The probabilities *p*_1_,…, *p*_*n*_ should sum up to 1, with an implicit “null” atom when the explicit probabilities sum up to less than 1.

Roughly speaking, for each clause containing a disjunction in its head, different instances are generated, each containing the clause with exactly one disjunct. The probability of a query would be given by the sum of all the probabilities of the instances whose models contain it.

We adopt the syntax of the *cplint* [45] implementation. Note that the disjunction in the head of clauses is indicated with the symbol “;”, while the conjunction is indicated as usual in Prolog with “,”. The equation in Figure 5 can be easily implemented with LPAD rules (Code 1 LPAD template with computed fall probability contributions):

fall(X) : c0.

fall(X) : c1 :- e1(X).

fall(X) : c2 :- e2(X).

...

Where c0≡*C*_0_, c1≡ *C*_1_, e1(X)≡ (*E*_1_=1), c2≡ *C*_2_, e2(X)≡ (*E*_2_=1) ...

The assessment tool should provide reliable information even when part of the subject’s data is missing. Missing values may arise when a test has not been (or cannot be) performed or the involved clinical professional does not consider its outcome decisive and reliable. In these cases, we have used the prevalence of the risk factors extending Code 1 as follows:

fall(X) : c0.

e1(X) : p1 :- u1(X).

fall(X) : c1 :- e1(X).

e2(X) : p2 :- u2(X).

fall(X) : c2 :- e2(X).

...

where u1(X), u2(X)… is true when the existence of the factor 1, 2… for subject *X* is not determined.

A scalar factor, with exposure levels from 0 (no exposure) to *m* (maximum exposure), is implemented similarly to a set of *m* dichotomous factors, one for each exposure level starting from level 1. The LPAD rule related to level *k* fires if the scalar risk factor has a level of *k* or higher.

Positive synergies (eg, comorbidities) between risk factors are well documented in the scientific literature. Since this would violate the causal independence assumption made before, we adjusted the model, following the Deandrea meta-analysis [29], introducing synergy factors.

A synergy factor, representing the potential synergies between *S* dichotomous risk factors, is implemented similarly to a scalar risk factor having a maximum possible level of *S* - 1 where, having a number of exposures equal to *q*, with 0 ≤ *q* ≤ *S*, the level is 0 if *q*=0 v *q*=1 and is *q* - 1 otherwise. So the risk starts increasing when there is a synergy between at least two factors.

#### Automatic Generation of the LPAD

The methodology that leads from risk factor odds ratio to LPAD rules is fully automatized. A working prototype has been produced and tested in the Java programming language (version 1.7); it may read risk factor odds ratios from an ontology or another source and outputs an LPAD program directly usable for risk assessment.

**Figure 11:**
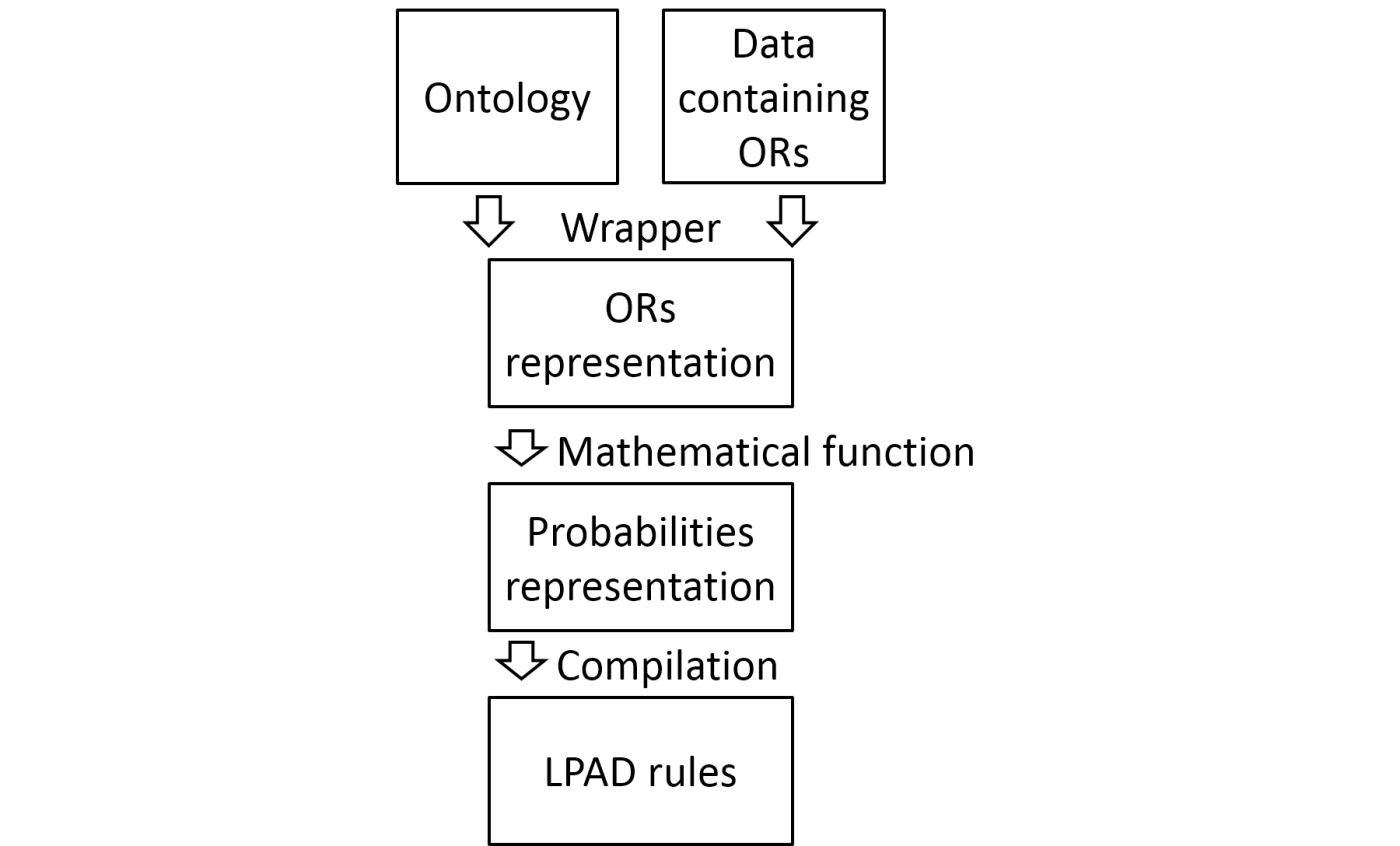
Steps in generating the LPAD rules.

Synthetically (see Figure 11), risk factors data complete with odds ratio are read from an ontology or other data source; a data structure containing odds ratios is created and then transformed (by means of the equation in Figure 9) in another containing probability values. Finally LPAD rules are compiled: these rules are applied to a subject to give their probability of falling in a given time span.

#### Dataset and Validation Procedure

FRAT-up discriminative performance and calibration have been tested on the InCHIANTI dataset (NCT01331512), where 1453 persons have been initially enrolled (1150 subjects aged 65 or more) and have undergone four consecutive visits globally covering a 9-year follow-up. It is a population-based epidemiologic study conducted in the Chianti region of Italy in two sites: Greve in Chianti (Area 1; 11,709 inhabitants; >65 years: 19.3%) and Bagno a Ripoli (Village of Antella, Area 2, 4704 inhabitants; >65 years: 20.3%). This study investigates age-related decline in mobility [46].

The InCHIANTI study started in September 1998 with the baseline assessment (first wave), which was completed in March 2000. Every 3 years, a follow-up assessment was performed. So, 3-year and 6-year follow-up assessments were performed respectively in 2001-2003 and 2004-2006 (second and third wave). A 9-year follow-up was then performed in 2007-2009 (fourth wave). The fifth wave is now ongoing.

At each wave, subjects were asked about the occurrence of any fall in the previous 12 months. In addition, clinical evaluation of the subjects was performed to collect information on fall-risk factors (other clinical variables were also collected, which are not of interest for this work [46]).

Our study used the information about risk factors from the first three waves, considering only subjects aged 65 or up. By doing so, we obtained 2319 samples from 977 subjects (every subject can have up to three samples).

At each wave, the risk factors of each subject were used prospectively to calculate their risk of falling at the subsequent wave (eg, the risk factors from the clinical evaluation at baseline were used to calculate the future risk of falling, which was compared with the recorded information on the occurrence of any falls in the 12 months before follow-up 1, and so on).

The estimators present in the InCHIANTI dataset and the algorithms to derive the risk factors from them are listed in Multimedia Appendix 1.

The discriminative ability and calibration of FRAT-up were validated by means of receiver operating characteristic (ROC) curve, area under the ROC curve (AUC), Brier score, and the Hosmer-Lemeshow test [47]. Since FRAT-up requires no training of the algorithm based on the available data, these metrics were computed by using all the available data as the test set.

## Results

**Figure 12:**
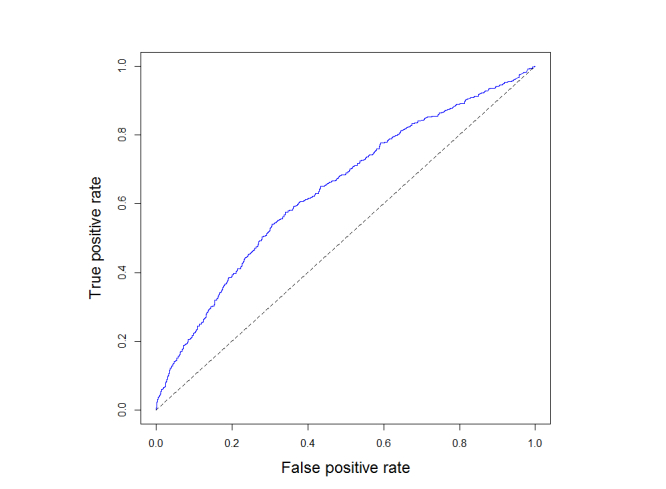
ROC curve obtained on the InCHIANTI dataset.

**Figure 13:**
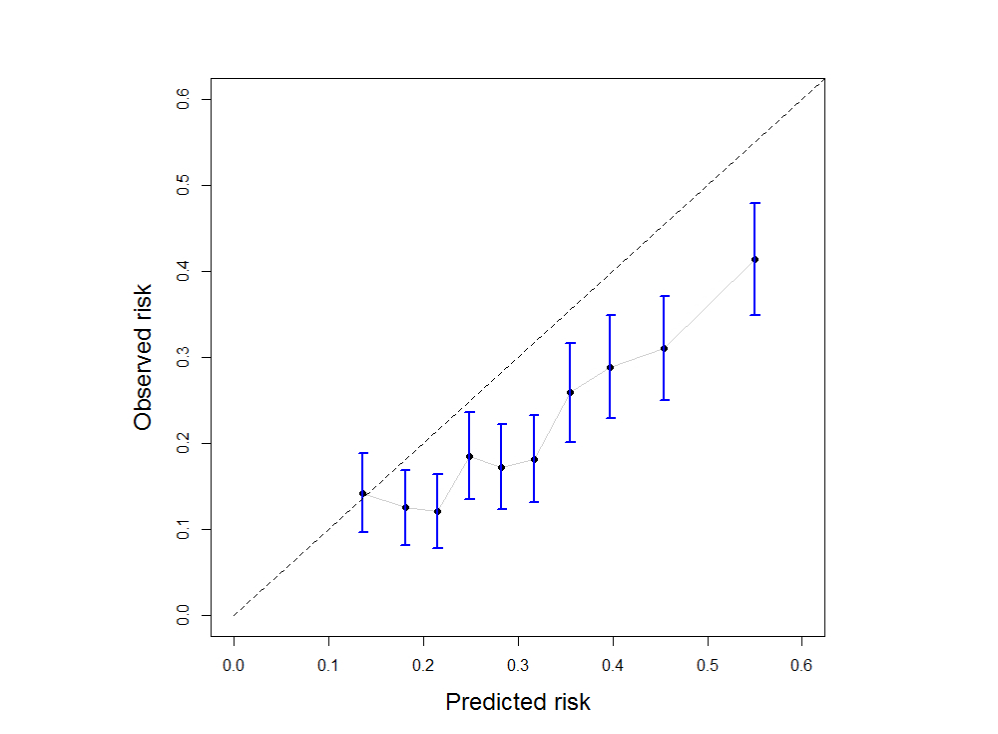
Calibration plot; sample (N=2319) used for validation where divided in 10 deciles, according to their predicted risk. For each decile, the mean predicted risk and the observed proportion of positive cases (proportion of fallers) are shown on the X and Y axes, respectively. Bars indicate 95% confidence intervals.

The ROC curve can be seen in Figure 12; the AUC value is 0.642 (95% CI 0.614-0.669). The Hosmer-Lemeshow test produces a very low *P* value (<.001) indicating statistical significance of miscalibration. As shown by the calibration plot in Figure 13, this miscalibration is due to risk overestimation that is consistent over the risk strata. The Brier score is 0.174.

## Discussion

### Principal Findings

The ability to discriminate between subjects who fall and subjects who do not fall, as operationalized by the AUC (0.642), compares favorably with other commonly used screening tools: a recent meta-analysis has estimated that the AUC of the TUG is between 0.54 and 0.59 [28], while the POMA-balance (also known as Tinetti balance scale) has AUC around 0.56 [23]. Since at each wave of the study, each subject was asked whether they had fallen in the last 12 months and the waves were about 3 years apart, this means we evaluated a prediction for an event that materializes between about 24 and 36 months after the assessment of the risk factors. Had the information about falls been available for the year just after the assessment, the results would likely have been better. Additionally, it is worth noting that the InCHIANTI dataset was not specifically designed to investigate fall risk. Because of these limitations, validation on other datasets would be desirable.

FRAT-up overestimates the fall risk. Since this overestimation, as shown in Figure 13, is consistent across deciles, the miscalibration is of less concern. The main reason behind this overestimation could be that the incidence of falls from [1] (31% subjects fallen at least once in a year), which was used for calculating the term *C*_0_, is higher than the observed incidence of falls in the InCHIANTI population (22%). A possible way to reduce overestimation would be multiplying the output by a constant, but we did not exploit this kind of learning on the dataset.

FRAT-up does exploit existing knowledge as it was built only from information derived from the literature, which was systematized in a meta-analysis. By doing so, it avoids overfitting and overoptimism, problems well known to affect predictive models [48].

Although the validation on the InCHIANTI dataset is based on a specific set of estimators, the architecture allows for the use of different estimators. The results of the validation have been obtained from the InCHIANTI dataset, where the percentage of missing values ranges from 0% on some variables (eg, sex and age) to 17% on vision impairment.

**Figure 14:**
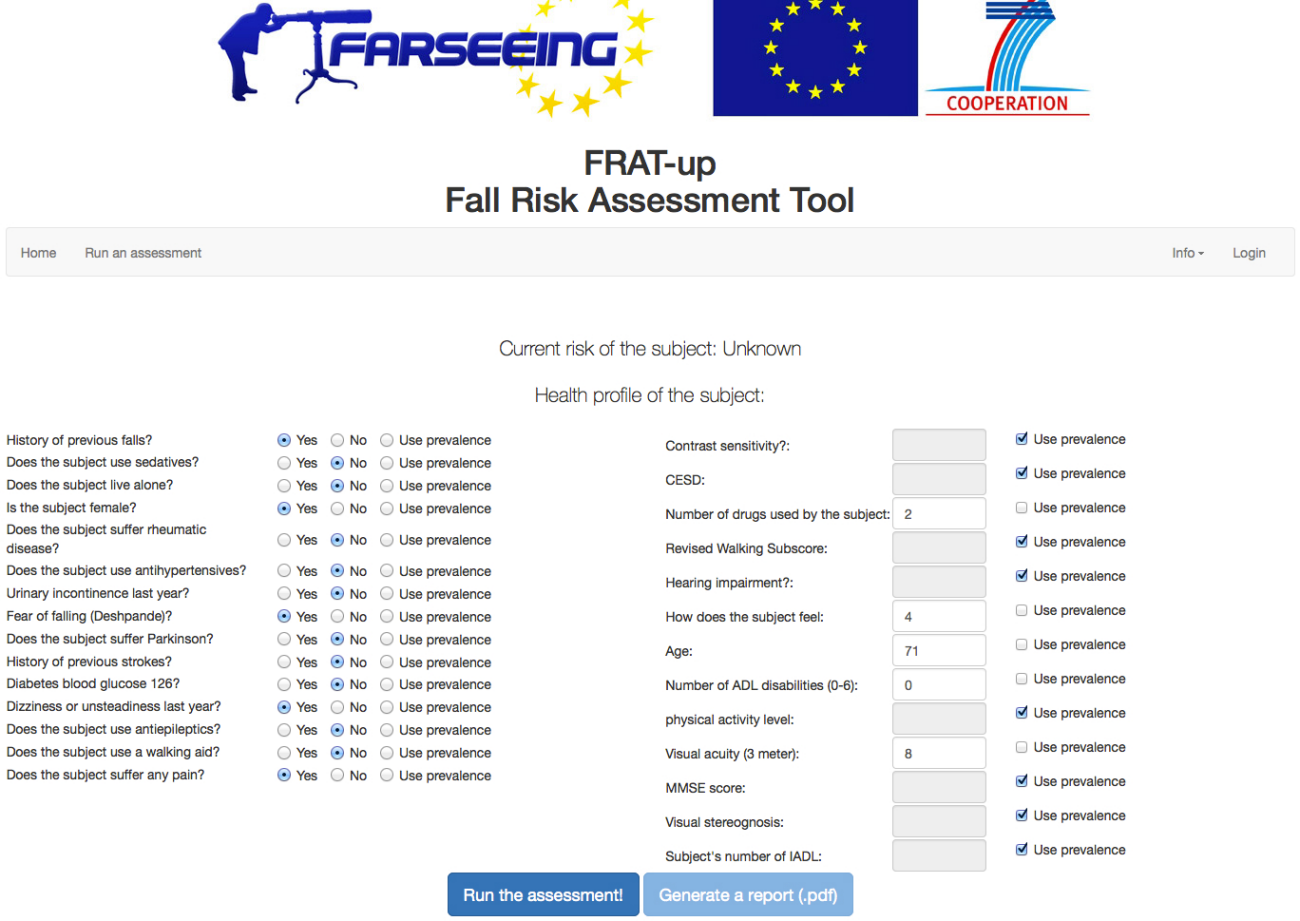
Screenshot of the Web-based interface.

The interactive prototype of the FRAT-up algorithm is freely available online [49]. Its interface is depicted in Figure 14.

### Limitations and Future Developments of FRAT-up

FRAT-up is based on the simplifying assumption that the risk factors contribute independently to the probability of falling. Following Deandrea et al [29] and to partially relax this assumption, we introduced the synergy factors in the methods section. However, different approaches may be investigated.

Our method showed robustness with respect to the missing values present in the InCHIANTI dataset. However, the extent to which the presence of missing values impacts the performance should be further investigated. In the future, FRAT-up will be tested on other datasets of different populations, possibly with different estimators for the risk factors, and compared with alternative risk assessment tools. Additionally, considering confidence intervals of the odds ratios could allow us in the future to assess the uncertainty associated with the fall-risk estimation.

Risk factors not reported in the meta-review by Deandrea et al [29] (such as rare risk factors) are not considered. Also, other information sources like experts’ opinion and administrative/demographic data are currently ignored. Ongoing work is devoted to extend the risk factor ontology with this additional information. The evaluation of the tool should go beyond statistical assessment alone. Usability and usefulness, which are increasingly acknowledged as important in the literature of prognostic models [50], will hence be evaluated.

Within the framework of a fall-prevention strategy, information would be useful on the indication of the modifiable risk factors of a specific subject and their quantitative impact on their risk. Practically, we foresee integration of the tool within electronic medical records, tools of general practitioners, as well as its adoption in public health bodies for population-wide evaluation.

The versatility of the presented solution will allow combining clinical information (that was used in this study) with other sources of data such as ambient sensor information or wearable sensors recording unsupervised long-term physical activity and/or quantitatively evaluating supervised or unsupervised physical performance by instrumented motor assessment [51-55].

An interesting extension of FRAT-up would be to implement it as an app for “smart” devices such as smartphones. The tool might be fed with rich sensor-based information and could be extended to provide “real-time” risk evaluation based on the subject’s current physical activity. Although from the technical viewpoint, such an extension would be easy and straightforward, using smartphone sensor data (in the fall-risk estimation) is still an open research issue.

Finally, since FRAT-up is based on a general methodology, it may be extended/applied in different ways, such as estimating fall risks in different settings (eg, acute care or nursing homes). Another extension would be to estimate outcomes other than falling, such as stroke risk, and more generally, estimate any risks directly related to the presence/absence of risk factors.

## Abbreviations

AUC: area under the ROC curve
FRAT-up: Fall-Risk Assessment Tool
LPAD: Logic Programming with Annotated Disjunctions
OR: odds ratio
POMA: Performance Oriented Mobility Assessment
ROC: receiver operating characteristic
TUG: Timed Up and Go Test

## Multimedia Appendix 1

FRAT-up estimators, factors, and procedures to produce factor values starting from estimator values.
