# Supplementary material for: FRAT-up, a Web-based Fall-Risk Assessment Tool for Elderly People Living in the Community
Source: J Med Internet Res. 2015 Feb 18;17(2):e41. doi: 10.2196/jmir.4064 (PMC4376110; doi:10.2196/jmir.4064)
Supplement: Supplementary file 1 [file jmir_v17i2e41_app1.pdf]

Multimedia Appendix 1 – Fall Risk Factors and Estimators

This appendix describes FRAT-up fall risk factors, estimators, and procedures to produce risk factor values starting from estimator values. There are two main types of estimators and risk factors. They are “dichotomous” (supporting missing data, so the possible values are “true”, “false”, and “unknown”) and “scalar” (integer values with the possibility of being unknown). Scalar values have a range [i,j], so a scalar [1,3] may take the values “1”, “2”, “3”, and “unknown”.

Risk factors

FRAT-up supports the same risk factors that were found to be significant in the Deandrea meta-analysis [1].

“Age” and “number of medications” are scalar risk factors. “Comorbidity” is a synergy risk factor. “Age” increases of a level every five years, starting from level zero at age 65 (e.g. it becomes level 1 at 70), with 4 as the maximum level for subjects aged 85 or more. “Number of medications” ranges from 0 to 10. For N=0,…,9, it takes level N on subjects taking N drugs. It takes level 10 on subjects taking 10 or more drugs.

Comorbidity counts the number of morbid conditions from the following list of 11 risk factors: cognition impairment, depression, diabetes, dizziness and vertigo, fear of falling, history of stroke, pain, Parkinson, poor self-perceived health status, rheumatic disease, and

urinary incontinence. More in particular, it works like a scalar risk factor with level 0 if there are 0 or 1 exposure, or level N-1 otherwise, with N being the number of morbid conditions. It thus can range from 0 to 10.

Fall risk factor prevalence from literature

In the following, the “Prevalence” column contains the probability to be “true” of Boolean risk factors (there are no “unknown” values since the following probabilities are extracted from scientific literature). The column contains the prevalence of the single levels for scalar risk factors, ordered from level zero upward.

Table b. Risk factor prevalence from literature, sources and notes

Name	Prevalence	Source	Notes
age	0.25, 0.25, 0.20, 0.16, 0.14	[2]	The distribution of age (divided in five years intervals) in the Italian population
cognition impairment	0.19	[3]	SPMSQ ≥ 3
comorbidity	Inferred		
depression	0.13	[4]	CES-D ≥ 16
diabetes	0.11	[5]	
dizziness and vertigo	0.20	[6]	
fear of falling	0.33	[7]	
female sex	0.48	[6]	
gait problems	0.42	[8]	Difficulty walking
hearing impairment	0.36	[4]	Questioning the participant on whether he/she could follow a conversation in a group of four persons (with a hearing aid if needed)
history of falls	0.31	[9]	People 65+ having fallen at least once in 12 months
history of stroke	0.13	[5]	
instrumental disability	0.37	[5]	One or more IADL impairment
living alone	0.32	[6]	

number of medications	0.237, 0.226, 0.194, 0.133, 0.081, 0.049, 0.036, 0.02, 0.01, 0.007, 0.007	[10]	Using medications in past two days, by number of medications, household population aged 65 or older, Canada excluding territories
Pain	0.30	[11]	
parkinson	0.008	[5]	
physical activity limitation	0.56	[12]	Self-reported physical activity levels in adults, by sex and age, England 2008, low activity: less than 30 minutes or more of moderate or vigorous activity on 1 to 4 days a week
physical disability	0.11	[3]	ADL ≤ 4
poor self perceived health status	0.20	[6]	Poor subjective health status (≥ 4)
rheumatic disease	0.47	[13]	Arthritis
urinary incontinence	0.19	[6]	
use of antiepileptics	0.01	[4]	
use of antihypertensives	0.32	[7]	
use of sedatives	0.14	[4]	Use of benzodiazepines
vision impairment	0.19	[4]	Questioning the participant on whether he/she could recognize someone's face at a distance of 4 meters (with glasses or contact lenses if needed)
walking aid use	0.18	[6]	

Estimators: types and notes

This section describes the estimators and how they are automatically extracted from the DB.

If an estimator value cannot be extracted from the DB for any reason, an “unknown” value is assigned.

Table d. Estimator types and notes

Name	Type	Notes
age	scalar [0,150]	
CESD	scalar [0,60]	[14]
contrast sensitivity	scalar [1,19]	
diabetes blood glucose 126	dichotomous	Suspected diabetes is included
dizziness or unsteadiness last year	dichotomous	
fear of falling Deshpande	dichotomous	total fear of falling ≥ 1, computed as in [15]
female sex	dichotomous	
hearing impairment 0 to 3	scalar [0,3]	
history of falls	dichotomous	Question: "Did you ever fall down in the last 12 months?"
history of stroke	dichotomous	
how do you feel 1 to 5	scalar [1,5]	
living alone	dichotomous	Checks if the subject declares to live with another person in one of the relevant questions.
MMSE plain	scalar [0,30]	[16]
number of ADL	scalar [0,6]	Washing face and arms; controlling urination and bowel movements; dressing and undressing; getting in and out of bed; eating (e.g., holding a fork, cutting food, drinking from a glass); using the toilet.
number of drugs	scalar [0,10]	
number of IADL	scalar [0,8]	Using the telephone; using public transportation; cooking a simple meal; doing light housework (e.g., doing dishes, light cleaning); doing heavy housework (e.g., washing windows, floor); taking medications correctly; managing home finances; shopping daily for basic necessities.
pain	dichotomous	Questions about generic pain (e.g.

		muscular cramps), pain at feet, stomach pain, chest pain, pain in legs, back pain, pain at hips or knees
parkinson	dichotomous	
physical activity level	scalar [1,7]	
revised walking subscore	scalar [0,10]	From a continuous 0-1 number, scales linearly between 0-10, and approximates to the nearest integer.
rheumatic disease	dichotomous	
sedatives	dichotomous	
urinary incontinence last year	dichotomous	
use of antiepileptics	dichotomous	
use of antihypertensives	dichotomous	
visual acuity 3 m	scalar [1,11]	
visual stereognosis	scalar [0,9]	
walking aid use	dichotomous	

Scalar estimators: descriptions and values

Table e. Scalar estimators descriptions and values

Estimator	Description	Values
CESD	CESD total score (0-60)	
contrast sensitivity	Contrast sensitivity (0.05-2.0). Result multiplied by 10 and discretized	
hearing impairment 0 to 3	Do you have any trouble hearing (TH)?	0. No
		1. Slight deafness
		2. Severe deafness
		3. Conversation impossible
how do you feel 1 to 5	How would you evaluate your current health? How do you feel now?	1. Very poor
		2. Poor
		3. Fair (so-so)
		4. Good
		5. Very good
MMSE plain	MMSE raw score (0-30)	
number of ADL	Number of ADL disabilities (0-6)	
number of drugs	Number of drugs	
number of IADL	Number of IADL disabilities	

physical activity level	Physical activity level last year	1. Hardly any physical activity
		2. Mostly sitting/some walking
		3. Light exercise 2-4 hrs/week
		4. Moderate 1-2 hrs or light >4 hrs/wk
		5. Moderate exercise >3 hrs/wk
		6. Intense exercise many times/wk
		7. Walks 5+ km/day, 5+days/wk, 5+yrs
revised walking subscore	Revised Walking Continuous Sub-score (0 - 1). Discretized on 0-10	
visual acuity 3 m	Visual acuity,3 meter (Monoyer 1/10-11/10)	1. 1/10 Monoyer's scale
		2. 2/10 Monoyer's scale
		3. 3/10 Monoyer's scale
		4. 4/10 Monoyer's scale
		5. 5/10 Monoyer's scale
		6. 6/10 Monoyer's scale
		7. 7/10 Monoyer's scale
		8. 8/10 Monoyer's scale
		9. 9/10 Monoyer's scale
		10. 10/10 Monoyer's scale
		11. 11/10 Monoyer's scale
visual stereognosis	Visual stereognosis, number of tests passed	
number of ADL	Number of ADL disabilities (0-6)	

Estimators to risk factors conversion

In the following table, the “estimators” column lists, for each risk factor, the one or more estimators involved in its value assignment. The “conversion” column specifies the

function used to get the risk factor given the estimators. There are four possible types of conversion.

- Direct: there is a single estimator and the risk factor takes the same value as the estimator.
- Threshold: An inequality against a fixed parameter is checked and if positive the risk factor is "true".
- Threshold and OR: there is a threshold check (with possibly different inequalities and threshold parameters) for each of the estimators; an OR of the results produces the risk factor value.
- Discrete levels: a function of this type has three parameters: "step 1 start", "step size", and "last step". An estimator value less than "step 1 start" produces the resulting value 0. At "step 1 start" the function maps to 1, and increases by 1 for each "step size" of the input estimator, up to a maximum of "last step".

Table e. Conversions from estimators to risk factors

**Risk factor**	**Estimators**	**Conversion**
Age	age	discrete levels (step 1 start: 70, step size: 5, last step: 4)
cognition impairment	MMSE plain	threshold (≤ 20)
depression	CESD	threshold (> 20)
diabetes	diabetes blood glucose 126	direct
dizziness and vertigo	dizziness or unsteadiness last year	direct
fear of falling	fear of falling Deshpande	direct
female sex	female sex	direct
gait problems	revised walking subscore	threshold (≤ 5)
hearing impairment	hearing impairment 0 to 3	threshold (≥ 1)
history of falls	history of falls	direct
history of stroke	history of stroke	direct
instrumental disability	number of IADL	threshold (≥ 1)

living alone	living alone	direct
number of medications	number of drugs	discrete levels (step 1 start: 1, step size: 1, last step: 10)
Pain	pain	direct
parkinson	parkinson	direct
physical activity limitation	physical activity level	threshold (≤ 2)
physical disability	number of ADL	threshold (≥ 1)
poor self perceived health status	how do you feel 1 to 5	threshold (≤ 2)
rheumatic disease	rheumatic disease	direct
urinary incontinence	urinary incontinence last year	direct
use of antiepileptics	use of antiepileptics	direct
use of antihypertensives	use of antihypertensives	direct
use of sedatives	sedatives	direct
vision impairment	visual acuity 3 m, visual stereognosis, contrast sensitivity	threshold and OR ([visual acuity 3 m ≤ 5, visual stereognosis ≤ 3, contrast sensitivity ≤ 16])
walking aid use	walking aid use	direct

References

1. Deandrea S, Lucenteforte E, Bravi F, Foschi R, La Vecchia C, Negri E. Risk Factors for Falls in Community-dwelling Older People: A Systematic Review and Meta-analysis. Epidemiology 2010;21(5):658–668.

2. Istituto nazionale di statistica. Statistiche ISTAT.

3. Capon A, Lallo D Di, Mastromattei A, Pavoni N, Simeoni S. Incidence and risk factors for accidental falls among general practice elderly patients in Latina, Central Italy. 2007;31(4):204–211.

4. Tromp a M, Pluijm SM, Smit JH, Deeg DJ, Bouter LM, Lips P. Fall-risk screening test: a prospective study on predictors for falls in community-dwelling elderly. J Clin Epidemiol [Internet] 2001 Aug;54(8):837–44. Available from: http://www.ncbi.nlm.nih.gov/pubmed/11470394 PMID:11470394

5. Stone KL, Ancoli-israel S, Blackwell T, Ensrud KE, Cauley JA, Redline S, et al. Actigraphy-Measured Sleep Characteristics and Risk of Falls in Older Women. 2008;168(16):1768–1775.

6. Gaßmann KG, Rupprecht R, Freiberger E. Predictors for occasional and recurrent falls in community-dwelling older people. Z Gerontol Geriatr 2009;42:3–10. PMID:18327690

7. Chu L, Chi I, Chiu A. Incidence and predictors of falls in the Chinese elderly. Ann Acad Med Singapore 2005;60–72.

8. Covinsky KE, Kahana E, Kahana B, Kercher K, Schumacher JG, Justice AC. History and mobility exam index to identify community-dwelling elderly persons at risk of falling. J Gerontol A Biol Sci Med Sci [Internet] 2001 Apr [cited 2014 Apr 1];56(4):M253–9. Available from: http://www.ncbi.nlm.nih.gov/pubmed/11283200 PMID:11283200

9. World Health Organization Department of Ageing and Life Course. WHO global report on falls prevention in older age. Geneva, Switzerland: World Health Organization; 2008. ISBN:9789241563536

10. National Health Information Council. National Population Health Survey: Health Institutions Component, Longitudinal (NPHS). 2007.

11. Pluijm SMF, Smit JH, Tromp E a M, Stel VS, Deeg DJH, Bouter LM, et al. A risk profile for identifying community-dwelling elderly with a high risk of recurrent falling: results of a 3-year prospective study. Osteoporos Int [Internet] 2006 Jan [cited 2013 Jun 7];17(3):417–25. Available from: http://www.ncbi.nlm.nih.gov/pubmed/16416256 PMID:16416256

12. Townsend N, Bhatnagar P, Wickramasinghe K, Scarborough P, Foster C, Rayner M. Physical activity statistics 2012. 2012. ISBN:9781899088072

13. Clough-Gorr KM, Erpen T, Gillmann G, von Renteln-Kruse W, Iliffe S, Beck JC, et al. Multidimensional Geriatric Assessment: Back to the Future Preclinical Disability as a Risk Factor for Falls in Community-Dwelling Older Adults. Journals Gerontol Ser A Biol Sci Med Sci 2008 Mar;63 (3):314–320.

14. Radloff LS. The CES-D Scale: A Self-Report Depression Scale for Research in the General Population. Appl Psychol Meas [Internet] 1977 Jun 1 [cited 2014 Jul 9];1(3):385–401. Available from: http://apm.sagepub.com/content/1/3/385.short

15. Deshpande N, Metter EJ, Lauretani F, Ferrucci L. Interpreting Fear of Falling in the Elderly : What Do We Need to. 2010;32(3):91–96.

16. Folstein MF, Folstein SE, McHugh PR. "Mini-mental state" A practical method for grading the cognitive state of patients for the clinician. J Psychiatr Res [Internet] 1975 Nov [cited 2014 Oct 27];12(3):189–198. Available from: http://www.sciencedirect.com/science/article/pii/0022395675900266
